# Esophageal Candidiasis in Two Dogs With Megaesophagus: A Case Report

**DOI:** 10.1111/jvim.70184

**Published:** 2025-06-26

**Authors:** Katie L. Anderson, Karen M. Tefft

**Affiliations:** ^1^ Department of Clinical Sciences College of Veterinary Medicine, North Carolina State University Raleigh North Carolina USA

**Keywords:** *Candida*, case report, esophagus, infectious esophagitis

## Abstract

Esophageal candidiasis is the most common cause of infectious esophagitis in human patients, but to date, this disease has not been reported in companion animals. A 16‐month‐old male intact King Shepherd dog and a 2‐year‐old female spayed German Shepherd dog were presented for evaluation of chronic regurgitation with diffuse megaesophagus identified on thoracic radiographs. In both cases, esophagoscopy disclosed diffuse, white fungal plaques, and brush cytology showed severe esophagitis with numerous yeast structures. Fungal cultures in both cases were positive for *Candida albicans* . The dogs were treated with fluconazole, which led to improvement or resolution of esophageal candidiasis. Esophagoscopy to evaluate for candidiasis should be considered in dogs with megaesophagus that either fail to improve on medical management or suffer an exacerbation of previously controlled signs.

**Abbreviations**
GERD: gastroesophageal reflux disease
Ig: immunoglobulin
LES: lower esophageal sphincter
RI: reference interval

## 1 Introduction

*Candida* is a ubiquitous yeast found throughout the skin, gastrointestinal, and urogenital tracts of both humans and dogs [1, 2, 3, 4, 5, 6, 7]. This commensal organism may become pathogenic in immunocompromised individuals or when the local tissue environment is altered to favor yeast overgrowth [8, 9, 10, 11]. In these individuals, infection with *Candida* spp. (candidiasis) can lead to clinically relevant morbidity and even mortality [5]. Early recognition of the disease and rapid initiation of antifungal treatment is crucial to prevent adverse outcomes [5].

In human patients, esophageal candidiasis is the most common cause of infectious esophagitis, with *Candida albicans* being the most commonly isolated organism [1, 12]. This disease causes substantial morbidity in immunocompromised patients as well as immunocompetent patients receiving antibiotics and acid suppressants [8, 9, 11]. Esophageal candidiasis also has been implicated as a predictor of poor survival in elderly patients [13]. In addition, chronic esophageal candidiasis is associated with the development of esophageal cancer [14, 15].

In dogs, *Candida* spp. have been linked to systemic infections [16, 17, 18, 19], as well as localized infections in the urinary tract [2, 10, 20, 21, 22], skin [23, 24], external ear canal [25], peritoneum [26, 27], cornea [28, 29], joint [30], and heart valves [31]. However, although *Candida* spp. are known to be commensal organisms in the gastrointestinal tract of dogs [3, 4, 6, 7], esophageal candidiasis has not been described in dogs. In this case report, we describe the diagnosis and treatment of esophageal candidiasis in two dogs with megaesophagus.

## 2 Cases

### 2.1 Case 1

A 16‐month‐old, male intact King Shepherd dog was presented to a specialty hospital for evaluation of chronic regurgitation and weight loss. The dog had been diagnosed with a diffusely dilated esophagus on thoracic radiographs at the referring veterinarian 3 months before. At that time, the dog underwent exploratory laparotomy for suspected obstructive foreign material, but no foreign material was identified. Jejunal biopsy samples had mild lymphoplasmacytic enteritis on histopathology. The intestinal biopsy sites subsequently dehisced and were repaired. Postoperatively, the dog was treated with enrofloxacin and amoxicillin‐clavulanic acid. The dog had not received treatment for esophageal dilatation nor had been treated with acid suppressants.

On physical examination at a specialty hospital, the dog was underconditioned (body condition score 3/9) and a prominent left prescapular lymph node was identified. The remainder of the physical examination was unremarkable. A CBC showed a monocytosis (1665/μL; reference interval [RI], 75–850/μL) and eosinophilia (1665/μL; RI, 30–1264/μL). A biochemistry panel showed mild hypoalbuminemia (2.5 g/dL; RI, 3–3.9 g/dL). A urinalysis showed well‐concentrated urine (USG 1.035) with no proteinuria noted on dipstick. Thoracic radiographs showed marked, diffuse esophageal gas dilatation, and abdominal ultrasonography showed mild left medial iliac lymphadenopathy and mild hepatomegaly. Cytology of fine needle aspirates of the left submandibular and left prescapular lymph nodes identified lymphoid hyperplasia with eosinophilic inflammation. A fecal floatation was negative for parasitic ova. A basal serum cortisol concentration excluded the diagnosis of hypoadrenocorticism, and a serum total thyroxine, trypsin‐like immunoreactivity, pancreatic lipase immunoreactivity, cobalamin, folate, and lead concentrations were normal. Acetylcholine receptor antibody testing was within RI.

**FIGURE 1:**
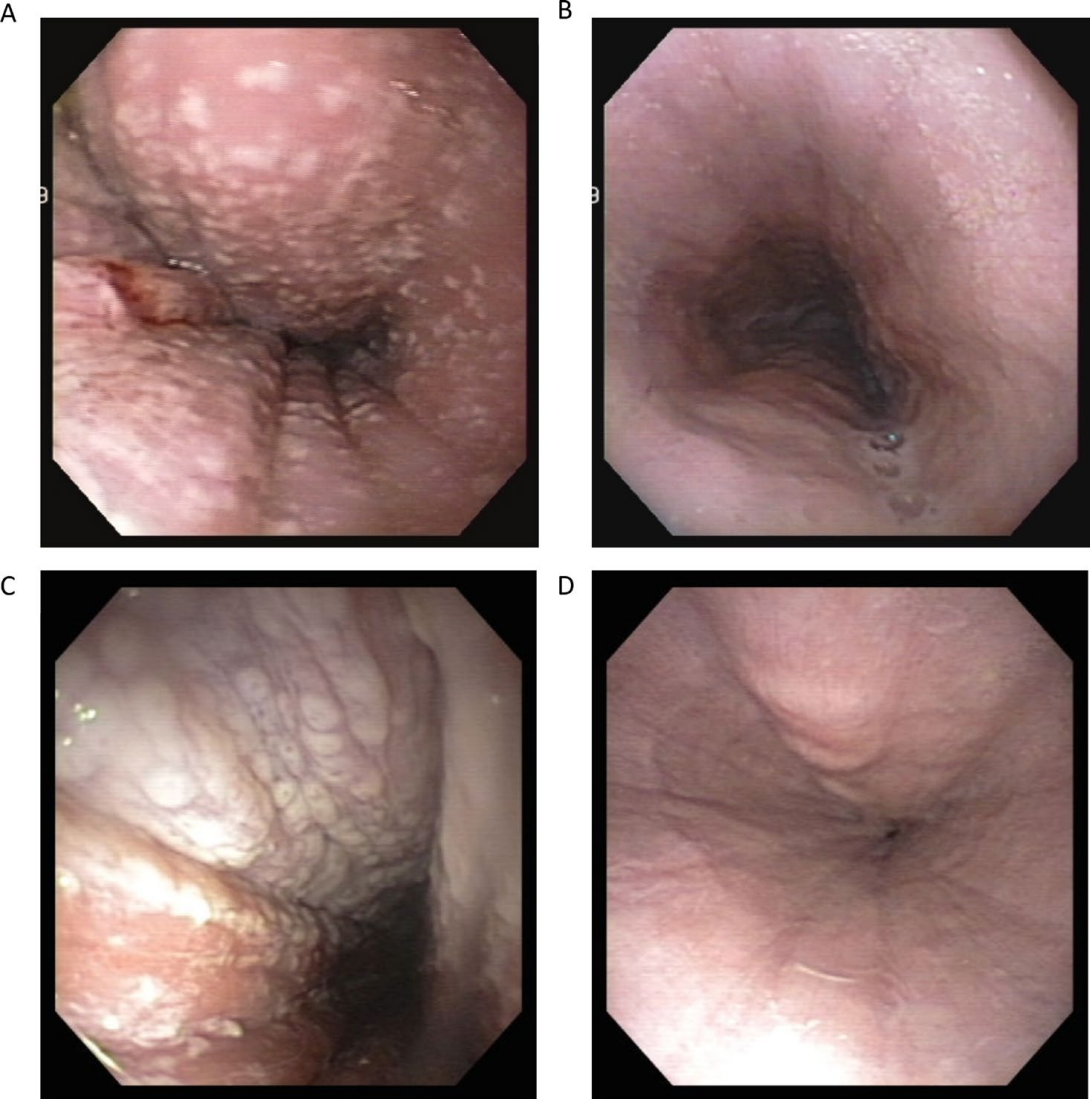
(A) Appearance of the esophagus in case 1 before fluconazole treatment. (B) Appearance of the esophagus in case 1 after 6 weeks of fluconazole treatment. (C) Appearance of the esophagus in case 2 before fluconazole treatment. (D) Appearance of the esophagus in case 2 after 11 weeks of fluconazole treatment.

**FIGURE 2:**
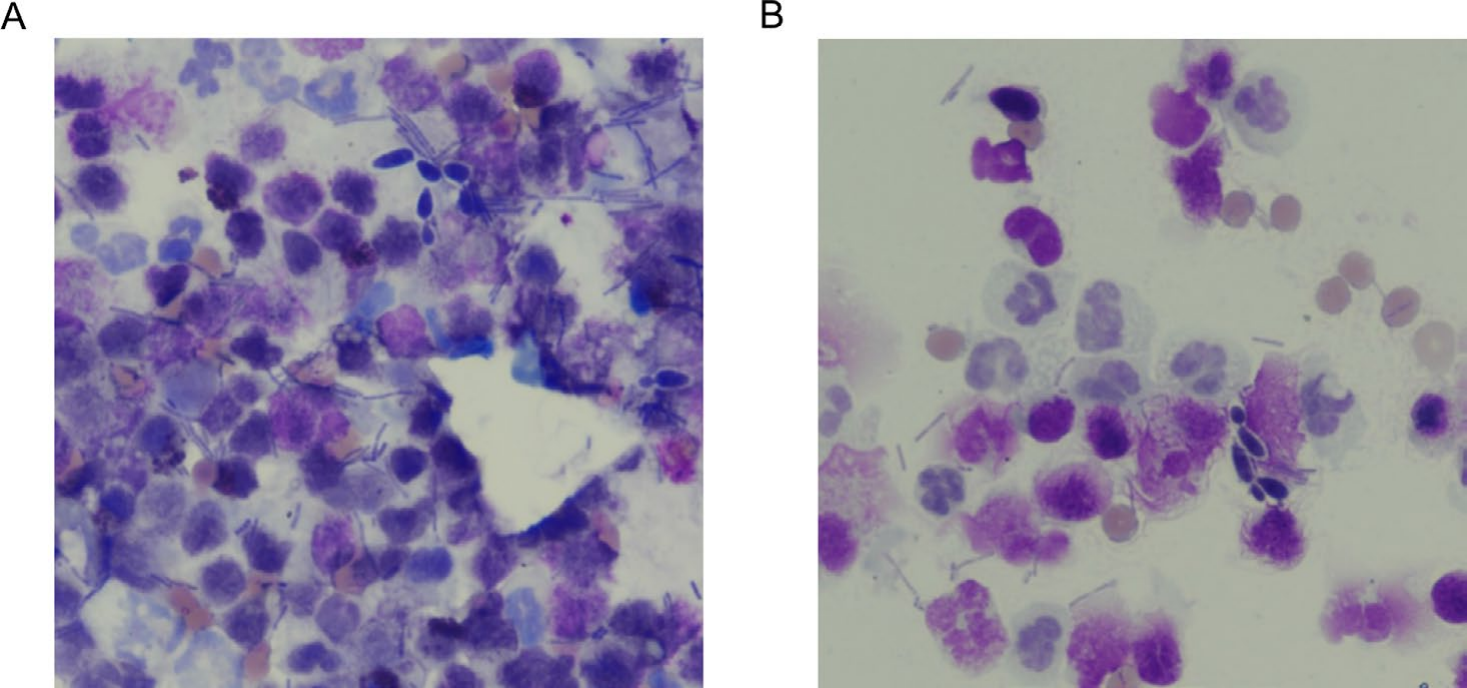
(A and B) Cytologic detection of septic suppurative inflammation with bacterial and fungal organisms, consistent with *Candida* spp., obtained from endoscopic brush cytology of the esophageal mucosa in case 1 (Wright‐Giemsa stain 50× magnification).

The dog was anesthetized for esophagogastroduodenoscopy. Endoscopy showed diffuse esophageal dilatation with severe, erosive esophagitis. Multifocal, punctate white plaques were noted diffusely, with increased numbers of plaques in the distal esophagus (Figure 1A). Brush cytology of the esophageal mucosa showed septic suppurative inflammation with intracellular rods and fungal organisms, consistent with *Candida* species (Figure 2A,B). Fungal culture of the sample grew *C. albicans* . Histopathology of esophageal biopsy samples showed diffuse, marked, erosive neutrophilic esophagitis with numerous intralesional bacterial rods. Periodic acid‐Schiff staining and Grocott's methenamine silver staining were negative for fungal organisms. The endoscopic appearance and histopathology of the stomach and duodenum were unremarkable. Given the dog's esophageal candidiasis, serum immunoglobulin (Ig) M, IgA, and IgG concentrations were evaluated and were within the RIs.

The dog was discharged on fluconazole (5.7 mg/kg PO q12h) for treatment of esophageal candidiasis and amoxicillin clavulanate (14 mg/kg PO q12h for 7 days) for treatment of suspected bacterial esophagitis. Sucralfate (1000 mg PO q8h), omeprazole (1.1 mg/kg PO q24h), and cisapride (0.57 mg/kg PO q12h) were prescribed for severe esophagitis and suspected gastroesophageal reflux disease (GERD). Upright feedings of a canned, low‐fat diet also were recommended.

The dog's regurgitation improved and 1 kg of weight gain was noted at a follow‐up visit. Repeat esophagoscopy was performed 3 weeks after the initial procedure and showed persistent diffuse esophageal gas dilatation with improved esophagitis and minimal *Candida* plaques. Fluconazole, cisapride, and omeprazole were continued at the previously prescribed dosages. A third esophagoscopy was performed 3 weeks after the second procedure and indicated improved to absent esophagitis with no visible *Candida* plaques (Figure 1B). Brush cytology of the esophagus identified septic suppurative inflammation with rare intracellular rods, but histopathology of esophageal biopsy samples was unremarkable. Fluconazole was continued for an additional 4 weeks and then discontinued. Omeprazole, cisapride, and upright feedings were continued long term. The dog's clinical signs remained well controlled over the next 2 years with minimal regurgitation.

### 2.2 Case 2

A 2‐year‐old, female spayed German Shepherd dog was presented to a specialty hospital for evaluation of chronic regurgitation that occurred primarily at night. Diagnostic tests performed by the referring veterinarian included a CBC, biochemistry panel, and serum total thyroxine concentration that were all within the RIs. The referring veterinarian prescribed sucralfate (1000 mg PO q12), famotidine (1 mg/kg PO q12), and a canned, low‐fat diet. The dog had not received treatment with antibiotics before referral.

At our referral hospital, physical examination was unremarkable. A biochemistry panel was within the RI apart from mild hyperglycemia (132 mg/dL; RI, 75–126 mg/dL) and hyperphosphatemia (5.6 mg/dL; RI, 2.6–5.3 mg/dL). Thoracic radiographs showed moderate, diffuse esophageal gas dilatation. An acetylcholine receptor antibody titer was within the RI. A basal serum cortisol concentration excluded the diagnosis of hypoadrenocorticism. The dog's regurgitation had resolved before referral, and the sucralfate, famotidine, and canned, low‐fat diet were continued as previously prescribed.

The dog's regurgitation recurred 1 month later despite ongoing medical management. A packed cell volume, total solids, and biochemistry panel were within the RIs. Thoracic radiographs disclosed persistent, diffuse esophageal gas dilatation. Treatment for GERD was initiated with cisapride (0.25 mg/kg PO q12), omeprazole (1 mg/kg PO q12), and famotidine (1 mg/kg PO before bedtime). A low‐fat, hydrolyzed diet also was prescribed and fed in an upright position. After these treatments were initiated, the dog's regurgitation markedly worsened. The regurgitation subsequently improved from multiple times per day to twice per week after discontinuing the cisapride and omeprazole.

Three months later, the dog was presented again for worsening regurgitation. Physical examination indicated 0.9 kg weight loss, decreased muscle condition score (2/3), and a dull, dry haircoat. Esophagogastroduodenoscopy identified diffuse esophageal dilatation with marked esophagitis. Diffuse, white, raised plaques were present, covering approximately 80% of the esophageal mucosa (Figure 1C). The lower esophageal sphincter (LES) was tightly closed and subjectively difficult to intubate. The gastric mucosa appeared mildly hyperemic with multifocal, white plaques present in the gastric body and antrum. Brush cytology of the esophageal mucosa identified suppurative inflammation with intracellular and extracellular rod bacteria and yeast structures, consistent with *Candida* species. Fungal culture was positive for *C. albicans* . Histopathology of esophageal biopsy samples was consistent with chronic, moderate to severe, erosive neutrophilic, eosinophilic, and lymphoplasmacytic esophagitis with rod bacteria. Histopathology of gastric and duodenal biopsy samples was unremarkable apart from low numbers of *Helicobacter* bacteria in the stomach. The dog was prescribed fluconazole (8.2 mg/kg PO q24) for esophageal candidiasis and marbofloxacin (4 mg/kg PO q24) for suspected bacterial esophagitis.

Repeat esophagoscopy was performed 3 weeks later because of a lack of improvement in the dog's regurgitation. Endoscopy identified persistent esophageal dilatation with diffuse, white plaques covering approximately 30%–40% of the esophageal mucosa. The LES again was tightly closed and difficult to intubate. Brush cytology of the esophagus identified septic suppurative inflammation with a predominately monomorphic population of intracellular and extracellular rod bacteria. Bacterial culture of the sample was positive for commensal organisms, including *Lactobacillus acidophilus* (2+), *Lactobacillus reuteri* (2+), and *Clostridium sporogenes* (2+). Given improvement in the dog's esophageal candidiasis, fluconazole was continued. Sildenafil also was prescribed (1 mg/kg PO q12) as a treatment trial for LES achalasia.

After starting sildenafil, the dog's regurgitation improved substantially and occurred only once in 3 weeks. Repeat esophagoscopy was performed 8 weeks after the second procedure and indicated improved esophagitis with white *Candida* plaques covering only 5%–10% of the esophageal mucosa (Figure 1D). Fluconazole was continued for an additional 4 weeks and then discontinued. Esophagoscopy was not repeated because of owner financial constraints. Sildenafil and upright feedings of a canned, low‐fat diet were continued. The dog's regurgitation remained well controlled for the next year.

## 3 Discussion

In this report, we describe the presence of esophageal candidiasis in two Shepherd dogs with megaesophagus. Although this disease is common in human patients, to our knowledge, it has not been described in dogs.

In human patients, esophageal candidiasis is common among immunocompromised patients, such as those with human immunodeficiency virus, diabetes mellitus, or transplant patients receiving immunosuppressive medications [1, 12]. The dogs in our case report included a purebred German Shepherd dog and a German Shepherd mix, and a subset of this breed has been shown to have mucosal IgA deficiency [32, 33]. Mucosal IgA has been shown in a mouse model to modulate *C. albicans* proliferation, preventing dysbiosis [34]. We were unable to test for a mucosal IgA deficiency in our patients, and thus it is unknown whether IgA deficiency or other immunodeficiencies contributed to the development of esophageal candidiasis in these dogs. However, immunodeficiency remains a possible cause. Among non‐immunosuppressed human patients, esophageal motility disorders and LES achalasia both have been identified as risk factors for esophageal candidiasis, along with the use of proton pump inhibitors and antibiotics [8, 11, 35, 36]. Both dogs in our study had megaesophagus, and one dog was suspected of having LES achalasia, given the response to sildenafil. Therefore, motility disorders likely contributed to the development of esophageal candidiasis in these patients. One dog had received treatment with antibiotics 3 months before the diagnosis of esophageal candidiasis, which also may have contributed to disease development.

Given that *Candida* spp. are commensal organisms in the gastrointestinal tract, isolation of *Candida* spp. alone is not sufficient to diagnose esophageal candidiasis [35, 37]. In human patients, a combination of clinical signs, endoscopic visualization of fungal plaques adhered to the mucosa, microbial culture of *Candida* spp., and cytologic or histopathologic evidence of esophagitis are used to definitively diagnose the disease [1, 35]. We utilized similar criteria to diagnose esophageal candidiasis in our patients. Specifically, endoscopic visualization of fungal plaques raised concerns for infection in our patients, which was confirmed with fungal culture and brush cytology. We recommend pursuing these diagnostic tests in megaesophagus patients with visible esophageal fungal plaques.

Systemic antifungal treatment is required for esophageal candidiasis in human patients [37]. Orally administered fluconazole is recommended, given its efficacy in clinical trials and lower cost [37, 38, 39, 40, 41], although itraconazole or voriconazole also may be used [38, 39, 40]. Posaconazole and amphotericin B are reserved for fluconazole‐refractory disease [37]. Fluconazole was utilized in both of our veterinary patients and effectively cleared the infection in dog 1, based on endoscopic visualization and brush cytology. Because of owner financial constraints, a fourth endoscopy was not performed in dog 2, and definitive clearance of esophageal candidiasis could not be confirmed. However, the infection appeared to be improving as of the third endoscopy, when the fungal plaques covered only 5%–10% of the esophageal mucosa compared with 80% of the mucosa at the time of diagnosis. This observation suggests that fluconazole might be an effective treatment for esophageal candidiasis in dogs.

Although esophageal candidiasis is typically a superficial infection in human patients, rarely it can lead to esophageal stricture formation, transmural necrosis, or esophageal perforation and sepsis [1, 12]. More commonly, the discomfort associated with esophageal candidiasis leads to decreased oral intake, weight loss, and malnourishment [1, 12]. Weight loss and malnutrition are commonly associated with megaesophagus in veterinary patients [42], and secondary esophageal candidiasis may exacerbate these problems. Therefore, treatment of esophageal candidiasis may be important to decrease morbidity in affected dogs with megaesophagus.

In conclusion, we describe the novel diagnosis and treatment of esophageal candidiasis in two Shepherd dogs with megaesophagus. Although esophageal candidiasis appears to be rare in dogs, early identification and treatment may lead to improved outcomes for affected patients. Clinicians may not routinely consider esophagoscopy in dogs with a radiographic diagnosis of megaesophagus because of the risk of aspiration pneumonia. However, these two cases illustrate that esophagoscopy to evaluate for candidiasis should be considered in dogs that either fail to improve on medical management or suffer an exacerbation of previously controlled signs.

## Disclosure

The authors declare no off‐label use of antimicrobials.

## Ethics Statement

The authors declare no institutional animal care and use committee or other.

## Conflicts of Interest

The authors declare no conflicts of interest.
